# Case-Control Study of Arsenic in Drinking Water and Lung Cancer in California and Nevada

**DOI:** 10.3390/ijerph10083310

**Published:** 2013-08-02

**Authors:** David C. Dauphiné, Allan H. Smith, Yan Yuan, John R. Balmes, Michael N. Bates, Craig Steinmaus

**Affiliations:** 1 School of Public Health, University of California, Berkeley, CA 94720, USA; E-Mails: dauphine@berkeley.edu (D.C.D.); ahsmith@berkeley.edu (A.H.S.); yanyuan@berkeley.edu (Y.Y.); jbalmes@medsfgh.ucsf.edu (J.R.B.); m_bates@berkeley.edu (M.N.B.); 2 Division of Occupational and Environmental Medicine, University of California, San Francisco, CA 94720, USA; 3 Office of Environmental Health Hazard Assessment, California Environmental Protection Agency, Oakland, CA 94720, USA

**Keywords:** arsenic, drinking water, lung cancer

## Abstract

Millions of people are exposed to arsenic in drinking water, which at high concentrations is known to cause lung cancer in humans. At lower concentrations, the risks are unknown. We enrolled 196 lung cancer cases and 359 controls matched on age and gender from western Nevada and Kings County, California in 2002–2005. After adjusting for age, sex, education, smoking and occupational exposures, odds ratios for arsenic concentrations ≥85 µg/L (median = 110 µg/L, mean = 173 µg/L, maximum = 1,460 µg/L) more than 40 years before enrollment were 1.39 (95% CI = 0.55–3.53) in all subjects and 1.61 (95% CI = 0.59–4.38) in smokers. Although odds ratios were greater than 1.0, these increases may have been due to chance given the small number of subjects exposed more than 40 years before enrollment. This study, designed before research in Chile suggested arsenic-related cancer latencies of 40 years or more, illustrates the enormous sample sizes needed to identify arsenic-related health effects in low-exposure countries with mobile populations like the U.S. Nonetheless, our findings suggest that concentrations near 100 µg/L are not associated with markedly high relative risks.

## 1. Introduction

Millions of people are exposed to arsenic in drinking water. In Bangladesh alone, >50 million people have been exposed to concentrations exceeding the World Health Organization guideline of 10 µg/L [1]. In 2001, the United States Environmental Protection Agency estimated that about 13 million people in the U.S. had public water supplies exceeding the new 10 µg/L regulatory limit [2]. More recent estimates suggest that over two million people in the U.S. may be exposed to concentrations >10 µg/L through private wells [3]. 

The International Agency for Research on Cancer has classified arsenic in drinking water as a known cause of human cancer of the skin, bladder, and lung [4]. Numerous studies have shown that the human lung is especially susceptible to ingested arsenic [5,6,7,8,9], and some data suggest that lung cancer is the most common cause of death from arsenic in drinking water [10,11]. 

The cancer risks associated with arsenic in drinking water, even at relatively low concentrations, may be quite high. An expert committee of the U.S. National Research Council estimated that lifetime exposure to arsenic at 10 µg/L may be associated with an excess risk of lung or bladder cancer as high as one case for every 300 people exposed [6]. These risks may be even higher in populations with early-life exposures [12], certain diets, genetic characteristics [13], or exposure to other carcinogens like tobacco smoke [14,15,16]. Importantly, these estimated risks, and the 10 µg/L arsenic standard, are based on linear extrapolations of cancer risks observed with concentrations above 200 µg/L. The risks from lower arsenic concentrations are unknown. This is important because most concentrations worldwide are <100 µg/L, and it is unknown if the arsenic-cancer dose-response relationship at lower levels is truly linear. Some authors have suggested that this might not be the case, and the current standard of 10 µg/L may be either overly or insufficiently protective [17,18]. This controversy, fueled by the high costs of removing arsenic from contaminated water, highlights the importance of clarifying health risks near or below 100 µg/L. 

The purpose of the present study was to investigate arsenic-associated lung cancer risks using a case-control study design in a population exposed to low to moderate arsenic levels in drinking water. This is the first lung cancer study on the largest U.S. populations with arsenic exposures between 50 and 100 µg/L [19], and the first study of lung cancer incidence in the entire U.S. with individual data on past drinking water arsenic concentrations [4,5,6,7,8].

## 2. Methods

### 2.1. Study Area

The study area consisted of six counties in western Nevada (Carson, Churchill, Douglas, Lyon, Mineral, and Storey) and Kings County in central California. In Churchill County, in the city of Fallon (population 8,600 in 2010), arsenic concentrations in public drinking water supplies were 85–125 µg/L from 1942 until 2004, when a treatment plant reduced concentrations to <10 µg/L [16,20]. In Hanford, California (population 54,000 in 2010), measurements averaged 110 µg/L in 1967, 90 µg/L in 1974, 68 µg/L in 1982, <40 µg/L in 2004, and <10 µg/L since 2009 [21,22]. A few other small towns in these counties had arsenic concentrations between 10 and 50 µg/L, and some private wells still exceed 100 µg/L, especially around Fallon. Most other water supplies in the study area had arsenic concentrations below 10 µg/L, offering a marked contrast in exposure [16]. 

### 2.2. Participant Selection

Inclusion criteria for lung cancer cases were: (1) Primary lung cancer first diagnosed between 2002 and 2005, (2) Histologic confirmation, (3) Over 25 years old when diagnosed, (4) Living in the study area at the time of diagnosis, and (5) Able to provide interview data (or having a next of kin or other close relative who could). In Nevada, lung cancer cases were ascertained using rapid case ascertainment, in which subjects were identified directly from each hospital in the study area as well as from hospitals in Reno, Nevada, the large metropolitan center neighboring the study area. In California, lists of subjects meeting inclusion criteria were provided by the Cancer Registry of Central California, which abstracted cases from all hospitals in Kings County and neighboring counties. Completeness of the Cancer Registry of Central California has been estimated at 95% [16]. Completeness of Nevada case ascertainment is estimated at 84% based on lung cancer incidence rates from the Nevada State Health Division for the years of this study [23].

Controls were selected using random digit dialing of home telephone numbers in the study area and frequency-matched to cases by 5-year age group, gender, and state (California or Nevada). At the time of control ascertainment, few people in the study area had cellular phones and >90% had home phones. Inclusion criteria for controls were: (1) Never diagnosed with lung or bladder cancer; (2) Over 25 years old; (3) Living in the study area at the time of first contact; and (4) Able to provide interview data (or having a close relative who could). All procedures were approved by the University of California, Berkeley institutional review board. All participants gave informed written consent. 

### 2.3. Interviews

Participants were interviewed over the telephone by a trained interviewer using a standardized study questionnaire. The closest relative (e.g., spouse or child) was interviewed for 93 of the 196 cases (47%), who were too ill to respond or deceased. Participants were asked to provide the locations of all residences occupied for six months or longer over their lifetimes, including street addresses when possible. For each residence, participants were asked about drinking water sources (private well, community supply, bottled water, or other) and filter use at the time they lived there.

Participants were also asked how many glasses of water and water-based beverages and foods (e.g., coffee, condensed soup) they typically consumed one year prior to the interview or diagnosis, as well as 20 and 40 years before. They were reminded of where they lived, what work they did, and other major events in their lives to help them recall past drinking water intake. Respondents were asked to estimate consumption of tap water and other fluids separately for home, work, and other places.

Questions regarding tobacco covered ages when smoking started and stopped, typical number of packs smoked per week, and exposure to secondhand smoke as a child and adult. Finally, participants were asked to describe all jobs held for six months or longer and their knowledge of contact with 17 specific chemical, occupational and environmental exposures associated with lung cancer, including asbestos, silica, fiberglass, and fumes [24]. Jobs were classified as low, possible, or high risk by researchers blinded to disease and exposure status of subjects, based on the extent of participant exposures and the degree of evidence linking specific exposures to elevated lung cancer risks.

### 2.4. Arsenic Exposure Assessment

All known previous residences in the study area were linked to drinking water arsenic concentrations by researchers blinded to case-control status. Arsenic measurements for all community-supplied drinking water and for thousands of private domestic wells within the study area were provided by the Nevada State Health Division and the California Department of Health Services. In all, we obtained over 7,000 arsenic measurements. For all large community water sources, records dated back 25 years or more. When historical records were unavailable, efforts were made to collect a water sample from the residence. Previous research showed that arsenic concentrations in study area wells were relatively stable over time, with Spearman correlation coefficients ranging from 0.84 to 0.94 in samples collected up to 20 years apart [25]. If a well could not be measured, the median of all wells within the same square-mile section (defined by the U.S. Public Land Survey) was used. If <2 wells existed in the section, the median of all wells within 3,000 m was used, except around Fallon, where arsenic concentrations were more variable. Here, the median of all wells of similar depth (either above or below 14 m) within 2,000 m was used. Other estimates for unmeasured wells were evaluated (e.g., the single nearest measured well, the mean or median of all wells within 500 to 5,000 m) but these did not improve predictions in analyses involving randomly selected wells with known arsenic concentrations. Researchers were blinded to case-control status when calculating these proxy estimates. For bottled water and exposures outside the study area, arsenic levels were assigned values of zero. Water treated with a filter known to remove arsenic (e.g., reverse osmosis) was assigned 21% of the pre-filtered concentration [3].

### 2.5. Statistical Methods

Odds ratios were calculated using unconditional logistic regression because we used frequency matching rather than perfect 1:2 matching of cases and controls [26]. In the final analyses, each participant’s highest 1-, 5-, and 20-year average arsenic concentration was categorized as ≤10 (the U.S. regulatory standard), 11–84, or ≥85 µg/L (the level historically found in Hanford and Fallon). Recent research, mostly developed after this study was initiated, suggests arsenic-cancer latencies ≥40 years [12,27,28]. Therefore, analyses used various lag periods (e.g., excluding exposures less than 40 years before diagnosis (for cases) or interview (for controls)). Lifetime cumulative exposure was calculated by multiplying each residential arsenic concentration by the number of years at that residence, summed over all residences occupied by the participant. These were categorized as ≤0.1, 0.11–2,399, and ≥2,400 µg/L-years (equivalent to 30 years of exposure to 80 µg/L).

Arsenic intake (in µg/year) was estimated for each subject by multiplying residential arsenic concentrations by the amount of water reportedly consumed 1, 20, or 40 years ago (whichever was closest to the years the subject lived in the residence). Previous research has shown that intake of dietary variables including coffee and tea can be accurately recalled from the distant past [29], although similar data on water intake are not available. Odds ratios were similar whether or not drinking water intake data were used, so our primary analyses involved drinking water concentrations rather than intake.

In the final models, odds ratios were adjusted for sex, age (in 10-year age groups), smoking (categorized as never, moderate (<10 packs/week), or heavy (≥10 packs/week on average during period of regular smoking)), occupational or other exposure to known lung carcinogens (as dummy variables for possible or high risk), and education (dummy variable for beyond high school). Other variables, including continuous measures of smoking (e.g., pack-years), former smoking, secondhand smoke exposure, body mass index (BMI), income, and state (California or Nevada), had little impact on results and were therefore not included in final models. Student’s t-tests were used to compare the means of continuous variables. All *p*-values are two-tailed. All analyses were done in SAS version 9.2 (SAS Inc., Cary, NC, USA).

## 3. Results

The names of 312 lung cancer cases were received from the cancer registry and hospitals in California and Nevada. Of these, 45 could not be located (and thus could not be confirmed to have lived in the study area), and 27 were ineligible because they did not live in the study area or could not provide an interview or next of kin interview due to illness or language issues. Of the remaining 240 cases, 44 (18%) declined to participate. Among 476 controls meeting inclusion criteria, eight could not be located after initially agreeing to participate, and 29 were ineligible due to illness or language issues. Of the remaining 439 controls, 80 (18%) declined to participate. This led to a final sample size of 196 cases and 359 controls.

Table 1 shows demographic characteristics of participants. As expected, lung cancer cases and controls were similar in terms of variables used for frequency matching, including state of residence (OR = 1.18, 95% CI = 0.78–1.76) and gender (OR = 1.22, 95% CI = 0.86–1.74). The average age of cases was 70.2 ± 10.0 (mean ± SD), compared to 69.0 ± 8.6 for controls (*p* = 0.13). Cases had lower incomes and were less educated than controls. They were similar in terms of race. Only 3.6% of cases reported never smoking regularly, compared to 40.9% of controls (OR = 18.7, 95% CI = 8.9–44.3). Cases were also less likely to report exposure to other lung carcinogens and had lower BMIs, but these differences were small.

Table 2 shows drinking water characteristics of participants. Cases reported higher water intake (averaging 2.42 L/day compared to 2.12 L/day for controls) but spent slightly less time in the study area (34% of person-years preceding enrollment compared to 40% for controls). While living in the study area, cases were more likely to use public supplies (74% of person-years compared to 62% for controls). Controls were more likely to use bottled water (10% *versus* 6% for cases) and private wells (29% *versus* 19% for cases). Most bottled water had low arsenic concentrations (<10 µg/L). Out of 293 wells reportedly used by participants in the study area, we measured or found records for 75 (measured between 1980 and 2010). We used proxy measurements or estimated concentrations for 87, which accounted for 6% of total person-years in the study area for cases, and 7% for controls. The remaining 131 wells, classified as unknowns, accounted for 8% and 10% of total person-years in the study area for cases and controls, respectively. Concentrations in wells ranged from non-detectable to 1,460 µg/L (median = 7 µg/L, mean = 36 µg/L). A similar percentage of cases and controls had 5-year average arsenic concentrations ≥85 µg/L at least 40 years before enrollment and cumulative exposures ≥2,400 µg/L-years.

**Table 1:** Demographic characteristics of lung cancer cases and controls, western Nevada and central California, 2002–2005.

			Cases (n = 196) No. %		Controls (n = 359) No. %		Crude OR	95% CI
*State* *								
	Nevada		146	74.5	278	77.4	1.00	0.78, 1.76
	California		50	25.5	81	22.6	1.18	
*Gender* *								
		Females	106	54.1	212	59.1	1.00	
		Males	90	45.9	147	40.9	1.22	0.86, 1.74
*Income*								
		<$20,000 per yea	50	25.5	65	18.1	1.00	
		$20–80,000 per year	105	53.6	214	59.6	0.64	0.41, 0.99
		>$80,000 per year	14	7.1	55	15.3	0.33	0.17, 0.66
		Don’t know or refused	27	13.8	25	7.0	1.40	0.73, 2.71
*Education*								
		High school or less	149	76.0	210	58.5	1.00	
		More than high school	47	24.0	149	41.5	0.44	0.30, 0.66
*Race*								
		Caucasian	182	92.9	323	90.0	1.00	
		Hispanic	4	2.0	17	4.7	0.42	0.14, 1.26
		Other	10	5.1	16	4.5	1.11	0.49, 2.50
		Unknown	0	0.0	3	0.8	--	--
*Smoking*								
		Never smoked	7	3.6	147	40.9	1.00	
		<10 packs/week **	117	59.7	160	44.6	15.4	6.94, 34.0
		≥10 packs/week **	72	36.7	52	14.5	29.1	12.6, 67.2
*Other lung carcinogen* ***								
		No	164	83.7	271	75.5	1.00	
		Yes	32	16.3	88	24.5	0.60	0.38, 0.94
			Mean	SD	Mean	SD	*p*-Value	
Age (years) *			70.2	10.0	69.0	8.6	0.13	
Body Mass Index (kg/m^2^)			24.6	4.3	25.9	4.4	<0.001	

CI confidence interval, OR odds ratio, SD standard deviation. * Participants were frequency matched on age, sex, and state; ** Average packs of 20 cigarettes (or equivalent for cigars and pipes) usually smoked per week during period of regular smoking, if ever smoked >6 months; *** Possible exposure to known lung carcinogens including radon, asbestos, silica, fumes and smoke.

**Table 2:** Drinking water characteristics of lung cancer cases and controls, western Nevada and central California, 2002–2005.

			Cases	Controls	*p*-Value
*Average tap-water intake* (L/day)					
	Current		2.24	2.04	0.20
	20 years ago		2.84	2.35	0.03
	40 years ago		2.17	1.96	0.27
	Total		2.42	2.12	0.05
*Person-years inside study area* (%)					
	Lifetime before enrollment		33.6	39.9	<0.01
	<40 years before enrollment		50.2	59.0	<0.01
	≥40 years before enrollment		11.2	13.0	<0.01
*Water source* (% of person-years) **					
	Public		74.4	61.5	<0.01
	Bottled		5.6	9.6	<0.01
	Private well		19.0	28.5	<0.01
		*Available records or measurements*	5.1	11.6	<0.01
		*Proxy measurement*	6.2	7.0	0.06
		*No record or measurement*	7.7	9.9	<0.01
	Other		0.8	0.3	<0.01
	Unknown		0.4	0.0	-----
*Water source ≥40 years before enrollment* (% of person-years) **					
	Public		77.0	59.5	<0.01
	Bottled		4.9	12.4	<0.01
	Private well		18.1	28.1	<0.01
		*Available records or measurements*	8.0	15.2	<0.01
		*Proxy measurement*	2.4	5.9	<0.01
		*No record or measurement*	7.7	7.0	0.61
	Other		0.1	0.0	-----
	Unknown		0.4	0.0	-----
*Arsenic concentration* (%) ***					
	Highest 5-year average: 10-year lag				
		≤10 µg/L	71.9	67.1	
		11–84 µg/L	18.9	22.8	
		≥85 µg/L	9.2	10.0	
		Average concentration (µg/L)	24.7	23.3	0.87
	Highest 5-year average: 40-year lag				
		≤10 µg/L	86.2	87.5	
		11–84 µg/L	7.7	7.5	
		≥85 µg/L	6.1	5.0	
		Average concentration (µg/L)	16.6	9.9	0.40
	Cumulative exposure: 10-year lag				
		≤0.1 µg/L-years	35.7	31.5	
		0.11–2,399 µg/L-years	58.2	64.6	
		≥2,400 µg/L-years	6.1	3.9	
		Average exposure (µg/L-years)	263.4	142.2	0.38

* Person-years inside the study area divided by total person-years lived. ** Person-years for each source divided by person-years in the study area. *** Percent of subjects in each category, adjusted for filter and bottled water use.

A supplemental file shows smoking and drinking water characteristics for each of the 30 subjects with arsenic concentrations ≥85 µg/L (median = 110 µg/L, mean = 173 µg/L) at least 40 years before enrollment. Twenty-four subjects (80%) were exposed through the public supply of Hanford, California, which was estimated to have concentrations of 110 µg/L until 1970. Three (10%) were exposed through the public supply of Fallon, Nevada, which was estimated at 90 µg/L. Three more (10%) had concentrations >110 µg/L (all private wells near Fallon, Nevada), the highest being 1,460 µg/L. 

Table 3 shows lung cancer odds ratios for various categories of arsenic exposure, with 10- and 40-year lags, for all subjects. Odds ratios with 5- and 20-year lags (not shown in tables) were lower than those with 40-year lags (e.g., 0.68, (95% CI = 0.34–1.37) and 0.76 (95% CI = 0.36–1.60), respectively, for highest 5-year average arsenic concentrations ≥85 µg/L). Odds ratios were also near 1.0 with 10-year lags. For highest 5-year average concentrations ≥85 µg/L lagged 40 years and cumulative exposures ≥2,400 µg/L-years, adjusted odds ratios were above 1.0, but 95% confidence intervals were wide (OR = 1.39, 95% CI = 0.55–3.53 and OR = 1.20, 95% CI = 0.45–3.22, respectively). In analyses confined to smokers (Table 4), corresponding adjusted odds ratios were slightly higher (OR = 1.61, 95% CI = 0.59–4.38 and OR = 1.26, 95% CI = 0.45–3.56), but confidence intervals still included 1.0.

**Table 3:** Odds ratios and 95 percent confidence intervals for arsenic in drinking water and lung cancer in western Nevada and central California, 2002–2005.

		Cases	Controls	Unadjusted			Adjusted *		
				OR	95% CI	*p*	OR	95% CI	*p*
*Highest 5-year average: 10-year lag*									
	≤10 µg/L	141	241	1.00			1.00		
	11–84 µg/L	37	82	0.77	0.50–1.20	0.25	0.75	0.45–1.25	0.27
	≥85 µg/L	18	36	0.85	0.47–1.56	0.61	0.84	0.41–1.72	0.63
*Highest 5-year average: 40-year lag*									
	≤10 µg/L	169	314	1.00			1.00		
	11–84 µg/L	15	27	1.03	0.53–1.99	0.92	0.84	0.40–1.79	0.66
	≥85 µg/L	12	18	1.24	0.58–2.63	0.58	1.39	0.55–3.53	0.48
*Cumulative exposure: 10-year lag*									
	≤0.1 µg/L-years	70	113	1.00			1.00		
	0.11–2,399 µg/L-years	114	232	0.79	0.55–1.15	0.22	0.75	0.48–1.15	0.18
	≥2,400 µg/L-years	12	14	1.38	0.61–3.16	0.44	1.20	0.45–3.22	0.71

CI confidence interval, OR odds ratio. * Adjusted for age, sex, education, smoking history (never, moderate (<10 packs/week), or heavy (≥10 packs/week)), and possible exposure to another known lung carcinogen.

Odds ratios were similar when highest 1-year averages were used instead of highest 5-year averages, when next of kin data were excluded, and when proxy measurements for wells were replaced with zeroes, the concentration in the closest measured well, or the median or mean arsenic concentrations of all wells within various distances of the unmeasured well, as described in Section 2.4. A clear relationship between arsenic and lung cancer risk was still not apparent when lower exposure categories (e.g., <0.1, 0.1–10, and >10 µg/L) were evaluated. When arsenic exposure was entered as a continuous variable (either as the highest five-year average concentration or as cumulative exposure), odds ratios (e.g., for each 1 µg/L increase in arsenic concentration), were all near 1.00 and not statistically significant, even with 40-year lags (data not shown). Odds ratios for exposures less than 40 years before enrollment were also near 1.00 and not statistically significant, for either smokers or all subjects.

**Table 4:** Odds ratios and 95% CIs for arsenic in drinking water and lung cancer among ever smokers in western Nevada and central California, 2002–2005.

Cases Controls				Unadjusted			Adjusted *		
				OR	95% CI	*p*	OR	95% CI	*p*
*Highest 5-year average: 10-year lag*									
	≤10 µg/L	136	148	1.00			1.00		
	11–84 µg/L	35	47	0.81	0.49–1.33	0.41	0.73	0.43–1.23	0.24
	≥85 µg/L	18	17	1.15	0.57–2.33	0.69	0.90	0.42–1.92	0.79
*Highest 5-year average: 40-year lag*									
	≤10 µg/L	164	187	1.00			1.00		
	11–84 µg/L	13	18	0.82	0.39–1.73	0.61	0.66	0.30–1.44	0.30
	≥85 µg/L	12	7	1.95	0.75–5.08	0.17	1.61	0.59–4.38	0.35
*Cumulative exposure: 10-year lag*									
	≤0.1 µg/L-years	69	72	1.00			1.00		
	0.11–2,399 µg/L-years	108	133	0.85	0.56–1.29	0.44	0.68	0.43–1.06	0.09
	≥2,400 µg/L-years	12	7	1.79	0.67–4.81	0.25	1.26	0.45–3.56	0.66

CI confidence interval, OR odds ratio. * Adjusted for age, sex, education, smoking history (moderate (<10 packs/week) or heavy (≥10 packs/week)), and possible exposure to another known lung carcinogen.

## 4. Discussion

Overall, this study did not identify clear or markedly increased risks of lung cancer in people exposed to arsenic in drinking water at concentrations near 100 µg/L. The finding of higher odds ratios with longer lag periods is consistent with recent research suggesting an extended latency period for arsenic-related cancers [12,27,28]. In analyses focused on exposures 40 years ago or more, odds ratios for average exposures ≥85 µg/L were 1.39 (95% CI = 0.55–3.53) in all subjects and 1.61 (95% CI = 0.59–4.38) in smokers. Although odds ratios were greater than 1.0, these increases may have been due to chance given the small number of subjects exposed more than 40 years before enrollment.

There are several possible reasons why statistically significant increased risks were not identified in this study. It may be that concentrations near 100 µg/L are not associated with increased lung cancer risks in this population, or they are associated with increased risks too low to be detected at a statistically significant level in a study the size of ours. When this study was conducted, it was not evident that the latency between arsenic exposure and lung cancer might be much longer than that of tobacco, which is about 20 years [30]. In northern Chile, lung cancer rate ratios did not peak until at least 30 years after high exposures began [27] and remain very high (OR = 4.4, 95% CI = 2.6–7.4) nearly 40 years after high exposures ended [28]. Other studies have also found latency periods of 20 years or more [12,16,31,32].

Although arsenic-exposed areas comprised about 28% of the study population during subject ascertainment [33], only 9% of controls lived in these areas ≥40 years before interview. This high migration rate was also seen in the 2000 U.S. Census, where 35% of people in Fallon reported living outside the county just five years earlier. Overall, the small percentage of people who lived in the more exposed parts of our study area during the most relevant risk period (≥40 years before cancer diagnosis) contributed to the low power of this study, and can be a major limitation of other arsenic studies investigating associations with long latency periods.

Despite low power, the findings of our study are important for several reasons. First, they provide an example of the difficulties and tremendously (if not prohibitively) large sample sizes needed to identify arsenic-related health effects in low-exposure countries with mobile populations like the U.S. This highlights the importance that highly exposed countries like Taiwan, Bangladesh, India, and Chile have played in generating new information on arsenic [4,5,6,7,8,9,10,11,12,13,14,15,27,28]. Secondly, while this study was not large enough to help confirm or rule out increased risks of 40–60%, the confidence intervals for our effect estimates provide evidence that concentrations near 100 µg/L are not associated with markedly high relative risks (e.g., much above 3–4). Finally, the fact that the magnitudes of the odds ratios we identified are close to those predicted by extrapolations from high doses used by the U.S. Environmental Protection Agency and others to set drinking water regulations suggests that these extrapolations provide at least somewhat reasonable estimates of low-dose risks [6,8,34].

In our study, exposure classification was based on reported residences and the arsenic concentrations identified for those residences. Errors in assigning exposure may have occurred as a result of missing data, changes in arsenic levels in wells over time, use of proxy respondents, or inaccurate recall of past water intake. The impacts of most of these are expected to be small. For example, a previous analysis of arsenic concentrations in wells in the study area has shown that they remain stable over many years [25]. With regard to proxy interviews, in studies comparing responses of cancer cases and their next of kin, spouses were able to identify 70% or more of the residences reported by the cases [35]. Also, the percentage of cases and controls with missing or unknown water records was similar. Study subjects may have also been exposed to arsenic in food or water outside the study area. However, these exposures are unlikely to have caused major misclassification. Inorganic arsenic intake from food is generally a fraction of that from drinking water with an arsenic concentration of ≥85 µg/L [36]. Although drinking water concentrations ≥85 µg/L do occur in other parts of the U.S., they are relatively rare. In an assessment of arsenic levels including the U.S. Environmental Protection Agency’s Arsenic Occurrence and Exposure database, Frost *et al.* identified only one other county with arsenic levels similar to those historically seen in Fallon and Hanford (Jim Hogg County, Texas; population 5,109; estimated mean county arsenic concentration 77.9 µg/L) [19]. Although participants could not perfectly recall how much water they drank in the distant past, it is it is not surprising that odds ratios were similar whether or not these data were used, because arsenic concentration was a much more important determinant of exposure than water intake. For example, the difference between our upper and lower exposure groups in terms of water concentrations was 10–20 fold (<10 *versus* ≥85 µg/L), whereas differences in water intake were rarely more than 3-fold. Overall, because researchers assessed exposure similarly in all subjects and were blinded to case-control status, exposure misclassification was likely non-differential, biasing odds ratios towards the null [37]. As such, improved exposure assessment likely would have resulted in higher, not lower, odds ratios than those identified. Importantly though, given the relatively low odds ratios identified, and the small numbers of subjects in the exposed categories, correcting for this misclassification would most likely cause only small changes. For example, correcting for an exposure categorization sensitivity of 70%, using the methods described by Rothman and Greenland, would only increase the odds ratio of 1.39 we identified to 1.40 [38].

Odds ratios in this study changed only slightly with adjustment for smoking, known occupational lung carcinogens, income, BMI, and education, suggesting that, while these factors differed somewhat between cases and controls, they were not strongly related to arsenic exposure and therefore did not cause important confounding. Previous studies suggest that smoking and arsenic may act synergistically [14,15,16]. In this study, odds ratios were somewhat higher in analyses confined to smokers, but we did not have adequate statistical power to evaluate synergy because of the small number of nonsmokers. For example, there were 12 cases and seven controls among smokers, compared to 0 cases and 11 controls among nonsmokers with arsenic concentrations ≥85 µg/L at least 40 years before enrollment, and 0 cases and seven controls among nonsmokers with cumulative exposures ≥2,400 µg/L-years lagged 10-years. The low number of non-smokers overall made it difficult to evaluate the effects of secondhand smoke. Confounding from other factors like diet, occupation, or environmental exposures (e.g., radon) is possible, but there is little evidence that these factors were sufficiently related to both lung cancer and arsenic exposure to either cause or mask a substantially elevated arsenic-lung cancer odds ratio [39].

Although only histologically-confirmed lung cancer cases were requested from the cancer registry and hospitals in California and Nevada, data on histological subtypes were not collected. Two recent studies [15,40] suggest that ingested arsenic may cause squamous and small cell carcinomas more than adenocarcinomas or other subtypes. For example, a study in the northeastern U.S. by Heck *et al.* reported a nearly 3-fold increase in squamous and small cell lung cancer (OR = 2.75; 95% CI = 1.00–7.57) in participants with toenail arsenic concentrations ≥0.1137 µg/g, despite no increase in all lung cancer types combined [40]. However, given the very low exposures (mostly ≤1 µg/L in drinking water), the small sample size (21 cases in the high exposure group), the large effect of adjustments for race, education, other lung disease, fish consumption, BMI, and other factors (unadjusted odds ratio of 1.41), and the difficulty of interpreting arsenic toenail levels [41], further research is needed to confirm the findings of Heck *et al.*

## 5. Conclusions

Lung cancer odds ratios for arsenic concentrations in drinking water ≥85 µg/L (median = 110 µg/L, mean = 173 µg/L, maximum = 1,460 µg/L) more than 40 years before enrollment were greater than 1.0, but these increases may have been due to chance given the small number of subjects. Our findings suggest that concentrations near 100 µg/L are not associated with markedly high relative risks (e.g., much above 3–4). The small number of people remaining in exposed areas for the extended latency period of arsenic-related cancer shows the difficulties of investigating the health effects of arsenic in low-exposure countries with mobile populations like the U.S. and illustrates why nearly all major findings on the health effects of arsenic have come from populations with high exposures in Taiwan, India, Chile, and Bangladesh [4,5,6,7,8,9,10,11,12,13,14,15,27,28]. New research on arsenic, including evaluating the impacts of early-life exposures, assessing possible mechanisms, and investigating susceptibility related to genetics, epigenetics, diet, co-exposures like smoking, occupation, and other factors, may be best done, at least initially, in countries with high exposures.

## Abbreviations

CI: confidence interval
OR: odds ratio

## Supplementary Files

Supplementary File 1 Supplementary (PDF, 50 KB)
