# Supplementary material for: Case-Control Study of Arsenic in Drinking Water and Lung Cancer in California and Nevada
Source: Int J Environ Res Public Health. 2013 Aug 2;10(8):3310–24. doi: 10.3390/ijerph10083310 (PMC3774440; doi:10.3390/ijerph10083310)
Supplement: Supplementary File 1 — Supplementary (PDF, 50 KB) [file ijerph-10-03310-s001.pdf]

Case-Control Study of Arsenic in Drinking Water and Lung Cancer in California and Nevada

Table 1. Smoking and drinking water characteristics for 30 subjects with ≥85 ug/L arsenic in drinking water at least 40 years before enrollment, western Nevada and central California, 2002–2005.

Lung cancer	Smoked	Packs/ week*	CA	Public water ≥ 85 µg/L	Arsenic in drinking water: Highest 5-year mean (µg/L) ≥40 years before	>5 years before	<40** years before	Cumulative (µg/L-years) >5 years before	Years since ≥ 85 µg/L**	Years since > 10 µg/L**
1	1	21	1	1	110	110	106	1,752	29	0
1	1	21	1	1	110	110	106	1,592	29	0
1	1	20	1	1	110	110	83	1,337	27	0
1	1	18	1	1	110	110	110	1,572	27	0
1	1	14	1	1	110	110	106	1,449	29	0
1	1	11	1	1	99	110	110	563	34	9
1	1	7	1	1	110	110	3	896	44	42
1	1	7	1	1	110	110	88	1,287	35	14
1	1	7	1	1	110	110	110	1,947	28	2
1	1	4	1	1	110	110	8	1,027	58	58
1	1	2	1	1	110	110	110	988	29	0
1	1	7	0	0	1,460	1,460	1,460	27,314	0	0
0	1	14	1	1	110	110	3	470	53	52
0	1	11	1	1	110	110	102	1,135	30	0
0	1	7	1	1	110	110	102	1,734	30	0
0	1	4	1	1	110	110	102	1,284	34	0
0	1	3	1	1	110	110	110	1,596	28	0
0	1	14	0	1	90	90	4	349	65	65
0	1	7	0	0	150	200	20	490	40	0
0	0	0	1	1	88	110	102	971	30	0
0	0	0	1	1	110	110	102	1,518	30	10

Table 1. *Cont.*

					**Arsenic in drinking water**					
					**Highest 5-year mean (μg/L)**			**Cumulative**		
**Lung cancer**	**Smoked**	**Packs/ week***	**CA**	**Public water ≥ 85 μg/L**	**≥40 years before**	**>5 years before**	**<40** years before**	**(μg/L-years) >5 years before**	**Years since ≥ 85 μg/L****	**Years since > 10 μg/L****
0	0	0	1	1	110	110	30	332	54	0
0	0	0	1	1	110	110	39	875	78	0
0	0	0	1	1	110	110	36	460	41	0
0	0	0	1	1	110	110	3	482	42	42
0	0	0	1	1	110	110	66	1,206	37	14
0	0	0	1	1	110	110	110	1,229	27	10
0	0	0	0	1	90	90	4	508	54	54
0	0	0	0	1	90	96	96	748	10	0
0	0	0	0	0	690	690	25	3,719	64	0
Percent or Average										
Cases	100%	11.5	92%	92%	222	223	200	3,477	31	10
Controls	39%	3.3	72%	89%	140	144	59	1,061	42	14
p-Value *(2-tailed)*	0.0001	0.002	0.17	0.81	0.50	0.52	0.25	0.29	0.06	0.67

CA California, 1 yes, 0 no. *Average packs of 20 cigarettes (or equivalent for cigars and pipes) usually smoked during period of regular smoking, if ever smoked >6 months. ** Exposures lasting <5 years before enrollment were not counted.

© 2013 by the authors; licensee MDPI, Basel, Switzerland. This article is an open access article distributed under the terms and conditions of the Creative Commons Attribution license (http://creativecommons.org/licenses/by/3.0/).
